# Impact of climate changes during the last 5 million years on groundwater in basement aquifers

**DOI:** 10.1038/srep14132

**Published:** 2015-09-22

**Authors:** Luc Aquilina, Virginie Vergnaud-Ayraud, Antoine Armandine Les Landes, Hélène Pauwels, Philippe Davy, Emmanuelle Pételet-Giraud, Thierry Labasque, Clément Roques, Eliot Chatton, Olivier Bour, Sarah Ben Maamar, Alexis Dufresne, Mahmoud Khaska, Corinne Le Gal La Salle, Florent Barbecot

**Affiliations:** 1 OSUR-Géosciences Rennes, Université Rennes 1 - CNRS, 35000 Rennes, France; 2 BRGM, Laboratory Department, 3 av C. Guillemin, 45000 Orléans, France; 3 OSUR-ECOBIO, Université Rennes 1 - CNRS, 35000 Rennes, France; 4 Université de Nîmes, EA 7352 CHROME, 30021 Nîmes, France; 5 CNRS/UPS UMR 8148 IDES, Université Paris-Sud, 91400 ORSAY France; P.A. GEOTOP, Université du Québec à Montréal, Montréal Qc, H3C 3P8, Canada; 6 Aster Team Aix-Marseille Université, CNRS-IRD UM 34 CEREGE, Technopôle de l’Environnement Arbois-Méditerranée, 13545 Aix-en-Provence, France

## Abstract

Climate change is thought to have major effects on groundwater resources. There is however a limited knowledge of the impacts of past climate changes such as warm or glacial periods on groundwater although marine or glacial fluids may have circulated in basements during these periods. Geochemical investigations of groundwater at shallow depth (80–400 m) in the Armorican basement (western France) revealed three major phases of evolution: (1) Mio-Pliocene transgressions led to marine water introduction in the whole rock porosity through density and then diffusion processes, (2) intensive and rapid recharge after the glacial maximum down to several hundred meters depths, (3) a present-day regime of groundwater circulation limited to shallow depth. This work identifies important constraints regarding the mechanisms responsible for both marine and glacial fluid migrations and their preservation within a basement. It defines the first clear time scales of these processes and thus provides a unique case for understanding the effects of climate changes on hydrogeology in basements. It reveals that glacial water is supplied in significant amounts to deep aquifers even in permafrosted zones. It also emphasizes the vulnerability of modern groundwater hydrosystems to climate change as groundwater active aquifers is restricted to shallow depths.

The last glacial period resulted in drastic modifications of the hydrogeological regime^1^,^2^. Sedimentary basins show evidence of glacial recharge^1^,^3^,^4^,^5^,^6^,^7^,^8^. The case for fractured aquifers in basements is less clear. Modeling of the hydrogeological regime in the Fennoscandian and Canadian shield during the last 10,000 yrs, has also indicated potential phases of glacial groundwater circulation in the basement^9^,^10^. However, whether recharge of glacial water is continuous or not during the last glacial maximum is not known. Furthermore, relationships of recharge with permafrosted areas remain unclear as well as recharge mechanisms^11^,^12^,^13^.

In recent decades, basement saline fluids have been sampled at great depths (0.5–5 km) in the context of nuclear-waste disposal investigations, and other general research programs. There is now evidence that such fluids are relatively ubiquitous in basements worldwide^14^,^15^. However their origin remains difficult to decipher due to intensive water-rock interactions and the extremely long residence-times^13^. In several cases, analyses with geochemical tracers have suggested that the solutes are of marine origin^16^,^17^,^18^,^19^,^20^,^21^. There is however no clear evidence of a potential marine component in hard rock aquifers. As an example, some of the brines in the Canadian shield are suggested to be of Pleistocene cryogenic origin^22^,^23^ but are also suggested to be of marine origin, which would require a Devonian age^19^,^24^,^25^.

The mechanisms that could explain how and when marine fluids could be introduced into the basement remain poorly understood^26^,^27^. Furthermore, no knowledge of the mechanisms involved in the preservation of marine or glacial fluids and the time length of such preservation has been available until now.

Basement rocks also constitute major groundwater resources which have been little exploited. However, there is now growing pressure for more intensive use of these resources. In recent decades, anthropogenic pressure has resulted in major drawdown and chemical evolution^28^,^29^. Such pressure accentuates the modifications induced by modern global warming as temperature increase should limit groundwater recharge in a large part of the earth surface before the end of this century, although groundwater recharge should increase in more restricted areas^30^,^31^. Deciphering the effects of climate change on paleo-hydrogeological regimes in basements is therefore of great importance. More precisely, the effects and ages of marine transgression on fluid circulation in basements need to be reliably identified, and the velocity of solute leaching quantified, together with the effects and duration of glacial fluid circulation.

## Materials and Methods

### Geological and hydrogeological setting

**Figure 1:**
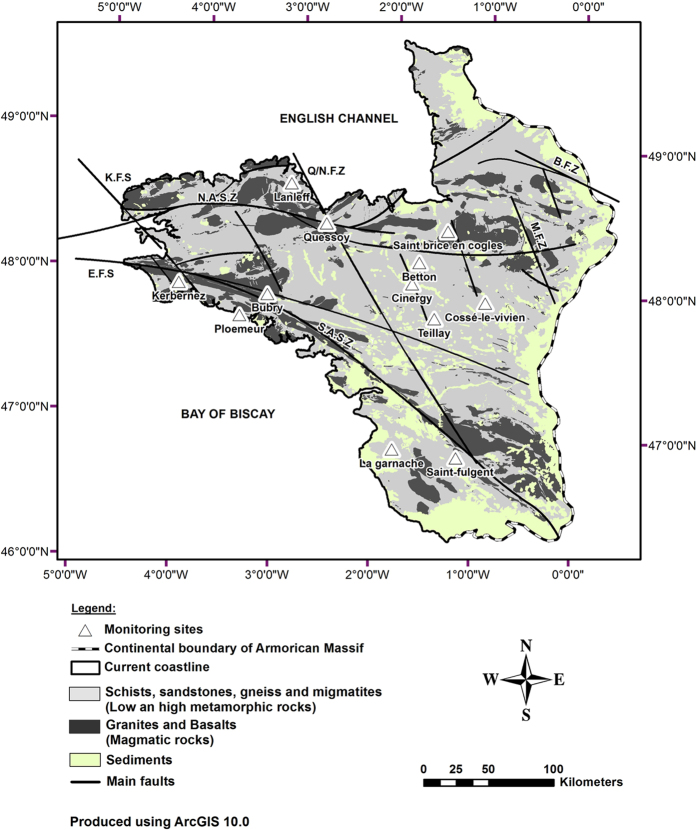
Location map of the sampling sites and geology of the Armorican Massif. Twelve sites showing high Cl concentration were chosen from the ADES national groundwater monitoring database. The well characteristics are presented in the Table S1. Two scientific monitoring sites (Kerbernez—Agrhys observatory and St Brice en Coglès) presenting shallow modern groundwater were also included to get a representative modern end-member. Three main geological domains are separated by large shear zones (South and North Armorican Shear Zones) and divided by more recent N140° fault zones (Elorn, Kerforne, Quessoy/Nort). The geological shapes have been manually created from detailed geological maps available on-line from BRGM (http://infoterre.brgm.fr). The coast line has been manually pointed as well as the sampling sites which have been pointed from their GPS coordinates.

The Armorican basement covers an area of 68,500 km^2^ in western France (Fig. 1). The Armorican Massif is a Variscan basement including some relics of the Cadomian orogenic belt^32^,^33^ (Fig. 1). The Armorican basement is made of three major units of Upper Proterozoic to Paleozoic formations separated by major N110E shear zones: the North Armorican Shear Zone (NASZ) and the South Armorican Shear Zone (SASZ). The Armorican basement is made of low and high metamorphic rocks (schist, sandstone, micaschist and gneiss), various plutonic rocks and locally some basaltic rocks. The Armorican basement was further densely fractured (N140E faults) during the Permian–Triassic extension. Some scattered remnants of Mesozoic and Cenozoic sediments are preserved on the Armorican basement. Although the Armorican basement has been thought as mainly continental during the Jurassic and Cretaceous times, it may have been at least partially flooded during this period^34^. During the Early Eocene global warming, the Armorican basement recorded the development of lateritic weathering several of meters thick, which was followed by semi-arid conditions (Late Eocene to Oligocene) until the Late Miocene cooling and the Mio–Pliocene boundary where the current climatic conditions prevailed across Europe^35^,^36^. During these periods, the Armorican Massif was flooded by rising sea-levels during the Early Eocene (Ypresian), the Late Eocene (Bartonian), the early Oligocene (Rupelian) and the Lower to Middle Miocene until about 11 M yrs^34^,^37^. According to stratigraphic records studies and to the eustatic sea-level fluctuations^38^, three main marine transgressions can be identified during most recent periods with three different paleocoastlines: (1) the oldest and the highest, is Mio-Pliocene (Messinian ~ 5.3+/−0.8 Myr) with sea level +90 m asl, (2) the second is Pliocene (Reuverian ~ 2.7+/−0.3 Myr) with sea level +60 m asl; and (3) the most recent is Pleistocene (Gelasian/Calabrian ~ 1.8+/−0.2 Myr) with sea level +30 m asl (Fig. S1 Supporting Information). Although the sea-level fluctuation curve shows three small events including the Pleistocene Gelasian/Calabrian one, sedimentological relicts seem to only characterize one of these events. As the Miocene events occurred more than 10 M yrs in the past, we focus in this study on the more recent Mio-Pliocene to Pleistocene events that are more likely the potential mechanisms for the observed salinization of the aquifers.

The altitudes of the Armorican basement reach 400 m in places but commonly range from 100 to 200 m. The climate is temperate. From a hydrogeological point of view, the weathered layer (0–30 m) constitutes an aquifer which is highly sensitive to diffuse agricultural pollution^39^. Additional aquifers can be found in the fractured rocks below this aquifer where water circulation is related to fault zones^40^.

More than 1,800 wells designed for groundwater exploitation are recorded in the ADES database for Brittany (http://www.ades.eaufrance.fr/). This database was used to analyze the spatial distribution of chloride concentrations in wells located further than 1 km from the coast line^41^.

Twelve sites in the area with very high chloride concentrations (>80 mg/L) were selected for geochemical investigation. Groundwater was collected after pumping in the deeper part of the aquifers below the weathered layer at depths of 50 to 150 m. One sample was collected from a deep research well (“Cinergy sample”) which intersected the basement from 405 m down to 675 m below a local sedimentary basin. This was the deepest and most saline sample (Cl = 1,208 mg/L). The samples collected, with Cl concentrations ranging from 74 to 144 mg/L, show evidences of mixing with modern and shallow groundwater (Cl < 50 mg/L). A sub-group of three sites (Bubry, Betton and Cinergy sites) with the most saline samples (Cl > 150 mg/L) is defined from PCA analysis (supporting information, section Sample origin and water-rock interaction). Below we compare these most saline fluids and other mixed samples to groundwater in the shallow weathered zone which contains modern and non saline groundwater. Although the number of saline samples is limited, it appeared clear from the chloride data base that very high chloride concentrations could be observed in various sites. The 12 sites were chosen in order to represent various geological formations as well as various places in the Armorican Massif (see sample location and characteristics Fig. 1 and Table S1, supporting Information). Although limited, this database is thought to be representative of the scale of the whole Armorican basement.

### Analytical methods section

Wells were pumped trying to install the pump in front of the most permeable fractures. Physico-chemical parameters (pH, Eh, T°C, Cond.) were monitored until stabilization was reached i.e. about 20 to 30 mn, usually 30 to 50% of the borehole volume. Samples were collected in polyethylene bottles rinsed with ultrapure water and acidified for cation analysis. Anions, cations and trace-elements were analysed in the Geosciences Rennes Laboratory using chromatography (anions) and ICP-MS (cations and trace).

Dissolved gases were collected without air contamination in steel bottles using a specific Grundfoss pump. CFC and SF_6_ were measured in the Rennes Condate-Eau analytical laboratory through gas-chromatographic analysis with ECD detector. Ne and Ar gases were measured though gas-chromatographic analysis with a catharometric detector. Uncertainty is about 3% for CFC and SF_6_ and about 5% for other gases.

All the isotopic analysis were carried out in the BRGM Laboratory. Water stable isotopes were determined through equilibration with δ^18^O and δD uncertainties of +/−0.1 and 0.5‰. Sulphate isotopic compositions were determined using a Delta S mass spectrometer (Thermo Finnigan). The δ^34^S of sulphates was measured from SO_2_ obtained from CdS precipitated after sulphate reduction and δ^18^O_-SO4_ was determined from the CO_2_ produced by the reaction of BaSO_4_ with C at 1050 °C. The Canon Diablo Troilite and Vienna-SMOW standards were used for S and O isotopes respectively with uncertainty about +/−0.3‰. Boron isotopes were determined by positive thermal ionization mass spectrometry on a Finnigan MAT 261 single collector solid source mass spectrometer. Values are reported on the *δ* scale relative to NBS951 boric acid standard. The long-term external reproducibility based on replicate analysis is ±0.4‰. The internal error is often better than 0.2‰

^36^Cl/^35^Cl ratio was measured by AMS at the French national ASTER facility. Chemical separation of Cl was conducted based on the protocol of Conard *et al.* (1986)^42^. To prevent S isobaric interferences, BaSO_4_ was precipitated by adding BaNO_3_ and was removed by filtration. Next, AgCl was precipitated by adding AgNO_3_ and recovered by centrifugation. To improve SO_4_ removal, the AgCl precipitate was dissolved in NH_4_ before repeating the procedure. AgCl is then precipitated again by adding NH_4_OH. A blank solution was prepared with Merck Standard NaCl 99.91%. The measured blanks provided ^36^Cl/^35^Cl ratios that were less than 10^−15^ at/at.

The carbon species (i.e. CO_2_, TDIC and carbonates) were converted into CO_2_ by direct acidification, and the ^13^C contents were measured by mass spectrometry (SIRA) at the IDES Laboratory (University of Paris Sud). The ^13^C content is reported using δ (‰) notation, as a deviation from the V-PDB (Vienna-Belemnite from the Pee Dee formation, North Carolina, USA). Graphite sources for ^14^C analyses were prepared from TDIC in the IDES Laboratory, and measured using accelerator mass spectrometry (UMS LMC14, Gif-sur-Yvette, France). The ^14^C contents are expressed as a percentage of modern carbon (pmC). Analytical errors, including laboratory errors, are of ±0.2‰ vs V-PDB for the δ^13^C, and between 0.1 and 0.3 pmC for the A^14^C.

## Results

### Solute origin

**Figure 2:**
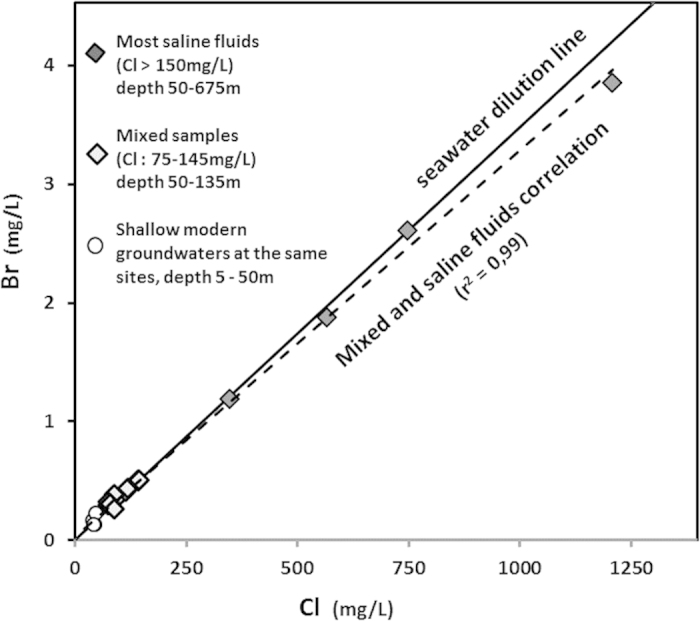
Bromide to chloride mass ratios. Three groups are distinguished on the basis of a PCA analysis (supporting information): (1) Shallow modern groundwater from the investigated sites and two monitoring sites, (2) samples showing mixing between deep saline samples and modern shallow groundwater, (3) three saline sites.

Whereas the Cl/Br mass-ratio range in the shallow and modern groundwater (<30 m depth) was very large (100 to 600) the Cl/Br mass-ratio of the saline fluids (Cl > 80 mg/L) collected in the Armorican basement was 283 +/−16 (Fig. 2). This ratio is very close to that of seawater (288). The linear Br to Cl relationship (R^2^ = 0.957) closely matches a dilution line ranging from the deepest, most saline fluid (Cinergy sample) to the most dilute shallow ones. This relationship is equal to the dilution line between the most dilute shallow samples and a modern seawater end-member. The boron isotope value for the more saline fluid (Cinergy) was +40.7‰, which is very similar to the isotopic composition of boron in seawater (+39‰). The boron isotope ratios in all the other saline fluids were close to this signature except in one sample where the ratios were lower, possibly as a result of interaction with clay minerals.

**Figure 3:**
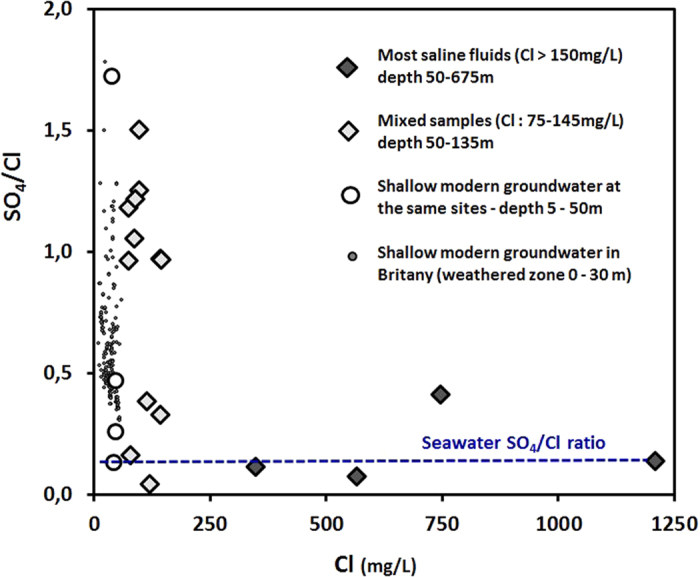
Sulphate to chloride mass ratios. A large modern groundwater data set (depth ranging from 5 to 25 m) from the Agrhys observatory (Kernernez site in Fig. 1) is presented.

Modern and shallow groundwater present a wide range of SO_4_/Cl mass-ratios (0.3 to 1.8 Fig. 3), generally far above the marine mass-ratio (0.14) and the SO_4_/Cl mass-ratio in local precipitation (0.44 +/−0.3). Oxidation of sulphide minerals such as pyrite, ubiquitous in igneous or metamorphic rocks increases the sulphate concentrations in the shallow groundwater. Furthermore, nitrate constitutes a major electron acceptor in modern agricultural catchments, which enhances sulphide oxidation whereas the oxidation mechanisms in groundwater that are not anthropogenically influenced are much more limited^43^,^44^. The sulphate concentrations in all the collected saline samples were high (mean concentration = 105 mg/L) with SO_4_/Cl mass-ratios ranging from 0.04 to 0.4 with a mean of 0.2 +/−0.14 when Cl concentrations is greater than 150 mg/L, close to the ratio found in seawater (0.22).

**Figure 4:**
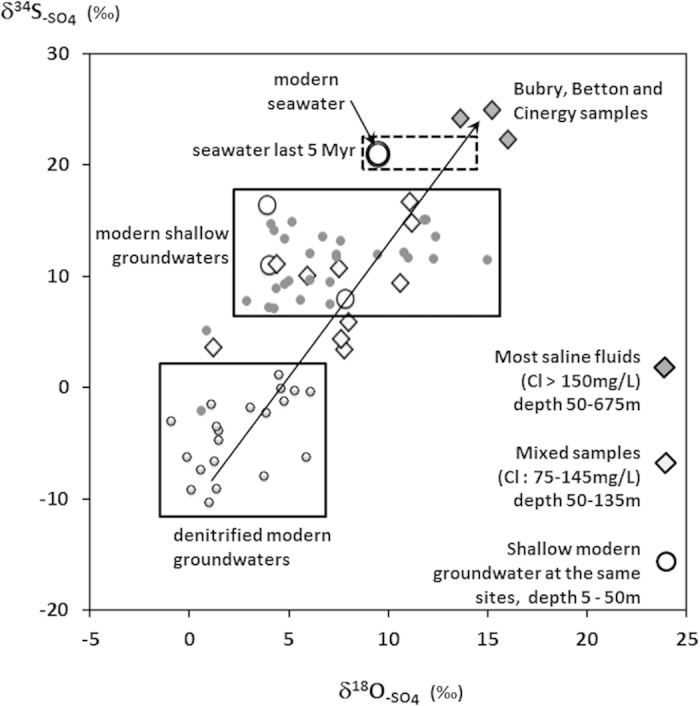
Sulphate isotopic ratios. A framework of interpretation is provided from previous data (Pauwels *et al.*, 2010). Two domains are identified: modern shallow groundwater and sites where autotrophic denitrification is assumed, i.e. groundwater showing low nitrate and high sulphate concentrations. Mio-Pliocene (Paytan *et al.*, 1998; Turchyn *et al.*, 2006) and modern seawater signature is presented. Arrow represents a potential mixing line between the denitrified groundwater, mixed groundwater and saline groundwater.

Different domains within the δ^18^O_-SO4_ vs δ^34^S_-SO4_ plot can be observed (Fig. 3). The δ^34^S_-_^_SO_^_4_ values for shallow groundwater, particularly nitrate-contaminated groundwater (i.e. NO_3_ > 5 mg/L), are within the range of +8 to +16‰. Groundwater influenced by sulphide dissolution present lower isotopic ratios (down to −10‰) as the isotopic ratios for sulphide δ^34^S_-SO4_ are usually negative^45^. δ^34^S_-SO4_ of the most saline fluids (Cl > 150 mg/l), ranges from +23 to +25‰. The other saline fluids can be plotted along a mixing line (arrow in Fig. 4) between two end-members: (1) a ^34^S depleted end-member containing sulphate originating from sulphide oxidations (denitrified fluids in Fig. 4) and (2) the fluids with the highest salinity. Both δ^34^S_-SO4_ (+22 to +25‰) and a δ^18^O_-_^_SO_^_4_ (+13 to +16‰) of the most saline fluid exceed that of present day seawater (around +21‰ and 9.5‰, respectively). Subsequent 0.8‰ and 6‰ decline of both δ^34^S_-_^_SO_^_4_ and δ^18^O_-_^_SO_^_4_ marine sulphate have taken place over the last 3 M yrs^46^,^47^, Therefore, the dataset indicates values for saline saline fluids slightly above those reported for 2 to 5 Myr seawater. However the slight difference can be explained either by a diagenetic reduction of sulphates within the first few centimeters of the sediment column, inducing a sulphate isotope fractionation or the occurrence of transitional environments such as lagoons and marshes.

### Chloride vertical distribution

**Figure 5:**
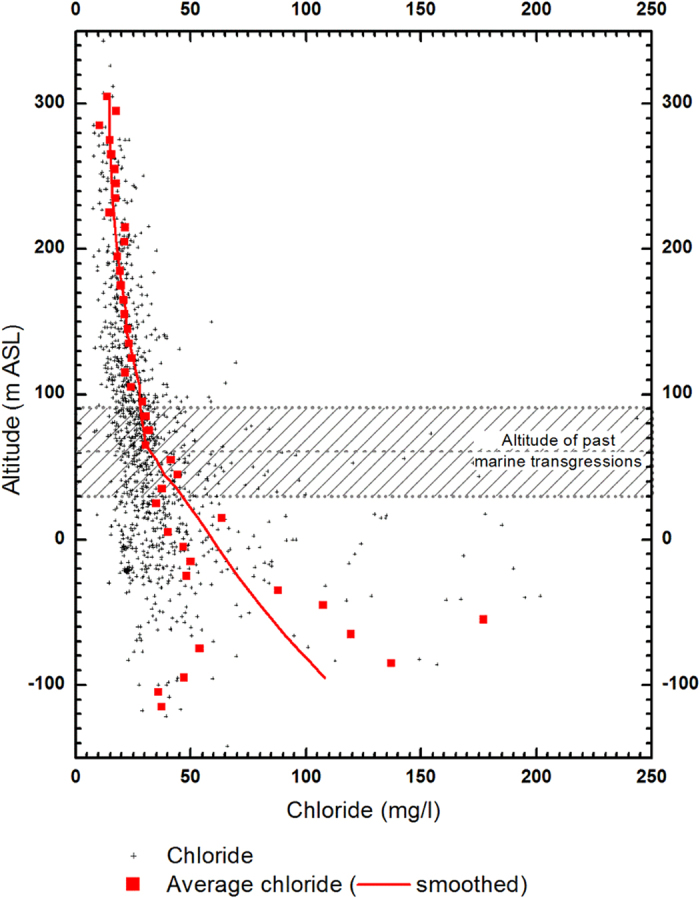
Vertical distribution of chloride concentrations. Chloride concentrations from 1,800 wells in the Armorican Massif recorded in the ADES national database are presented versus the well mean altitude above sea level. The paleo-coastlines of the three last transgressions are presented. A mean chloride concentration for each 5m layer is computed.

The chloride concentrations of all the Brittany wells in the ADES database have been plotted against altitude (asl) in Fig. 5. A 10 m depth-average is also presented (red curve). An increase of the chloride range and clear concentration increase with depth is observed, especially below 100 m asl. The mean chloride concentrations in the highest 250 m (i.e. above 100 m asl), vary between 15 and 30 mg/L. Such concentrations are indicative of meteoric and anthropogenic sources^41^. The sharp chloride increase observed below 100 m requires a third source. A more detailed investigation revealed that the chloride concentrations precisely reflect the last three transgression events^41^. This result strongly supports a marine origin for the water solutes below 100 m depth, whatever the site.

### Recharge temperature

The stable isotopes in the most saline fluids can all be plotted along the local meteoric water line (LMWL) and represent meteoric continental waters (Supporting information, stable isotopes section). Only the composition of the deepest and most saline Cinergy-sample differs from the LMWL. When possible, the most saline fluids were compared with shallow groundwater at the same location. A systematic shift towards more negative values was observed along the LMWL. Such a shift indicates that the recharge conditions were colder than modern ones. This shift is correlated to both the chloride concentration and the recharge temperature (Supporting information, stable isotopes and noble gas section). These correlations favor the temperature interpretation to other potential explanations (altitude, continental effect, cloud internal fractionation) although the extent of the shift is lower than expected from recharge temperatures. As atmospheric conditions may have evolved along with temperature from the last glacial maximum to modern conditions, the stable isotope shift is only used as a temperature indication.

Recharge temperatures were deduced, using an inverse model, from the Ar and Ne concentrations^48^ (supplementary information, stable isotopes and noble gas section). The recharge temperatures for the collected fluids ranged from 5 to 10 °C, except for the deepest and most saline Cinergy sample which presented a recharge temperature close to 0 °C and also extremely high excess air. The present-day recharge temperature in Brittany is about 12 °C and the noble gas measurements in shallow groundwater agree with such temperatures^40^. It is thus clearly apparent from the recharge temperatures that the recharge conditions were colder than today. This is particularly true for the deepest and most saline Cinergy sample, which would have required glacial or immediately post-glacial recharge conditions.

**Figure 6:**
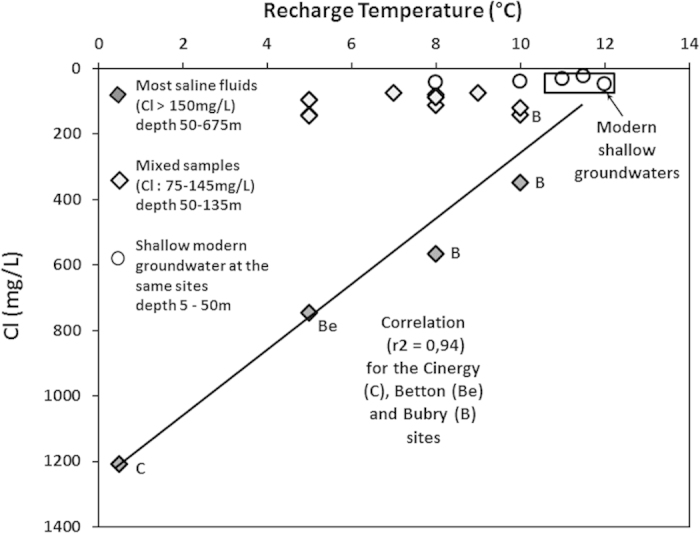
Recharge temperature deduced from noble gases vs chloride concentration. Paleo recharge temperature is deduced from the Ar and Ne content and is presented against the chloride concentration in the 12 sites investigated.

A clear correlation was observed between recharge temperature and chloride concentrations (Fig. 6). The three sites with most saline samples display a linear correlation between surface shallow groundwater and the deepest and most saline Cinergy sample.

### Groundwater dating

CFC, tritium and SF_6_ groundwater dating shows that modern groundwater presents residence times close to 20 yrs in the weathered part of the aquifers (0–30 m) and 20 to 50 yrs and more in the deeper part^33^,^40^ (with uncertainties +/−less than 2 yrs). All the saline fluids present CFC concentrations close to the detection limits which is interpreted as “old” fluids (>50 yrs) mixed with a limited amount of modern surface waters. On the basis of the nitrate and CFC content, the amount of modern surface fluids contained in the samples ranges from less than 1% to 15% (see supporting information for end-member used, mixing section and Table S2). ^36^Cl and ^14^C analysis were carried out within 3 samples including the two most saline ones (Cinergy sample Cl = 1,208 mg/L; Betton sample Cl = 700 mg/L). A detailed interpretation of these data is provided in supporting information (Section Groundwater dating, figure S2 and Tables S4 and S5). ^36^Cl in these samples is close to the equilibrium with the neutron production of the Proterozoic formations where the waters have been collected. This equilibrium process requires residence times of at least 10^6^ yr. Conversely, ^14^C indicates residence times of 17,100 to 17,600 yrs for the two most saline fluids. The ^36^Cl and ^14^C data are interpreted as representing two different events as presented below. The chemical basis for the scenario is also given in supplementary information (mixings section).

## Discussion

### A two-phase evolution

As already suggested by several studies of old, saline fluids, the solute and water may have different origins and represent different phases of fluid circulation corresponding to geologic or global climatic changes^15^,^16^,^19^. They will therefore be discussed separately. The agreement between chloride vertical distribution and the altitudes of the last transgressions, from 5.3 to 2 M yrs old, supports a marine origin of the solutes in groundwater below 100 m asl. High Cl concentrations, requiring a marine contribution, are observed throughout the Armorican basement except in the zones preserved from past transgressions at altitudes higher than 90 m asl where the Cl concentrations remain below 50 mg/L. This correlation is also supported by the geochemical relationships, Br/Cl and SO_4_/Cl mass-ratios, as well as the B and S isotopic ratios. The marine signature of the solutes from saline fluids in the basement thus represents the generalized influence of transgressions prior to the glacial period at the scale of the Armorican basement. ^36^Cl data that suggest secular equilibrium are in good agreement with the transgression timing.

It was concluded in several earlier studies of saline fluids worldwide that the solutes were of marine origin^16^,^17^,^18^,^19^,^20^,^21^. However it was difficult to confirm this due to intensive water-rock interactions. In contrast, this signature is clearly apparent in the Armorican aquifers for various solutes (Cl, SO_4_, Br, Cl/Br and Cl/SO_4_ ratios, B and S isotopic ratios). This may be related to the very short time since the last transgression (1.8 +/−0.2 M yrs).

On the contrary, the noble gases and water stable isotopic ratios clearly demonstrate that the water in these saline fluids is of meteoric origin and that recharge occurred during glacial or immediately post-glacial conditions. Glacial water strongly diluted the older seawater already contained in the basement rocks. We interpret this contradiction as reflecting a two-stage evolution namely: (1) Introduction of seawater-derived fluids into the basement 5.3 to 2 M yrs ago, which constitute the source of the solutes collected in most of the wells below 100 m asl in the Armorican basement. (2) Recharge of cold meteoric water. Glacial melt water introduction follows the end of the last glacial maximum 19,000 yrs ago at the onset of deglaciation of the permafrost in northwestern Europe 18 to 17,000 yrs ago^49^. Although it might be possible that glacial recharge has occurred several times during the past, following the various glaciation cycles, ^14^C ages mainly agree with a limited period at the onset of the last deglaciation.

**Figure 7:**
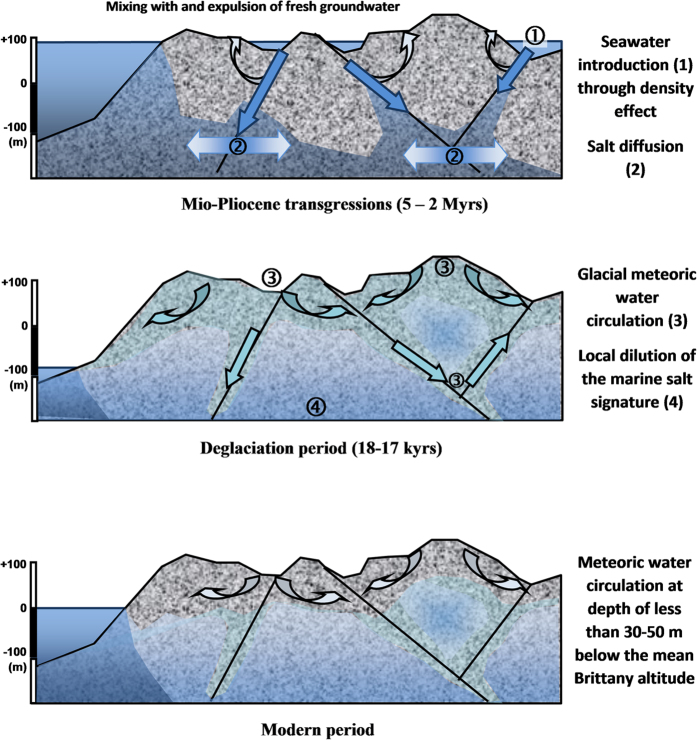
Hydrogeological model derived from the geochemical data. The hydrogeological model presents the three steps that have been established through the geochemical data. The first stage represents the introduction of seawater in the basement through density-driven process within major faults. Seawater is introduced in the lower parts of the basement where it is mixed with freshwater. Following introduction which is spatially focused, seawater is introduced in the rock matrix (micro-porosity) through a slow diffusion process. During the second stage, permafrost breakthrough induces the introduction of cold freshwater within the same major faults. This introduction is induced by large hydrogeological loops that occur probably during a relatively short period (less than a few thousand years). The third stage represents the modern period where the hydrogeological loops are less extended towards depth which allows for the preservation of the previous signatures below 100 m depth. Figure is original and has been drawned by L.A.

A hydrogeological model based on these observations is depicted in Fig. 7 and presents this two-phased circulation and the very different modern hydrogeological regime. These 3 situations are discussed below.

### Seawater introduction into the basement

The attribution of a marine signature to several deep saline samples worldwide may indicate that the mechanisms responsible for seawater introduction into basements occur in various places through geological time. The introduction of saline fluid into basements requires drivers. These may be upward fluxes related to basin brine expulsion. A typical example of such mechanisms is the fluid migration responsible for MVT-type metal deposits. However, such fluid circulation seems to be mainly restricted to the basin/basement interface^16^,^50^. As transgression occurs, the simplest mechanism of seawater introduction into the basement is by gravity- and density-driven displacement of former fresh groundwater by seawater. Such a process may account for saline fluid circulation at relatively great depth in the most permeable fractures. However, this mechanism cannot explain the relatively ubiquitous occurrence of saline fluids throughout the Armorican basement. Furthermore, the most saline fluids were collected in the Cinergy well at a depth of 405–750 m below a small Mesozoic sedimentary basin, away from the border faults of this grabben. These elements require that the process was thorough and efficient, allowing the saline fluid to penetrate all the scales of porosity and not only the major structures.

Diffusion may account for the penetration of marine fluid within the whole basement rocks. Molecular diffusion can be estimated from the diffusion of water molecules in water *D*_*m*_, which is about 10^−9^ m^2^.s^−1^ for typical groundwater temperatures. The diffusion length scale^51^ – i.e. the length of the mixed zone – is about 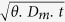, where *t* is the time since recharge, and *θ* is the porosity ranging from 0.01 to 0.1, leading to a mixing length of about 20 to 60m for 1 Myr, and from 2 to 6 m for 10 kyr. It would seem from these estimates that the marine chlorides are probably well mixed at the scale of the investigation, i.e. a few hundred meters. As seawater has been introduced into the basement at higher time-scales than the million years diffusion scale, it should have diffused throughout the hard rock porosity. Even the immobile water in the microporosity could contain marine solutes. This does not mean that the concentration is constant throughout the aquifer since (1) the flow is likely rapidly renewed in the upper part of the geological section, and (2) an increase in concentration, due to the density effect, should still be observed as the time required for complete homogeneous diffusion of the entire domain exceeds several M yrs. In particular, a depth gradient should be observed as the density-driven flow should result in a greater influence at depth.

### Glacial recharge

In contrast, glacial meteoric recharge presents the characteristics of a relatively intensive and short process. First, the deepest and most saline Cinergy sample contains extremely high excess air. Such excesses are thought to be characteristic of rapid recharge of glacial melt^3^,^52^, which would agree with the low recharge temperatures. This trapping of excess air implies a rapid recharge event. Second, the preservation of these glacial signatures in all the fluids investigated indicates a generalized process which has not been erased by further mixing with modern groundwater. Third, the Cinergy sample indicates that glacial fluids have penetrated at great depth which may occur as *i*) glacial groundwater has a high density and *ii*) seawater level increase is slower than permafrost melt. It is interpreted as a succession of rapid recharge events which occurred during a short period between 18 and 17,000 yrs ago after the glacial maximum. This period corresponds to the first retreat of northwestern glaciers and could also correspond to the breakthrough of the permafrost in Brittany^49^. Resumption of recharge processes have also been observed in sedimentary aquifers in northwestern Europe at a similar period^53^. During this period, the sea level was about 80m below its present level, which has allowed deeper penetration than the modern groundwater regime.

Hydrodynamic dispersion, which is the second process that might have diluted the original glacial or marine signal, is classically modeled by a diffusion equation. The diffusion coefficient in this equation is the product of the average flow velocity *v* by the hydrodynamic dispersivity length *A*. The dispersion length is thus equal to 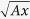, where *x* is the distance of flow since recharge. The distance from recharge zones is reasonably between 100 m and 1000 m for most groundwater samples. Hydrodynamic dispersivity values of between 10^−2^ and 10^3^ m have been reported in field scale studies but the reliability of values greater than 1 is poor^54^. The resulting estimated dispersion length is between 1 and 30 m. In contrast with molecular diffusion, the mixing length scale is only dependent on the flow distance and not on the time since the recharge event. This back-of-the-envelope calculation explains why we can have in the same place relatively high chloride concentrations, signatures of marine transgressions during warmer climates, as well as a glaciation recharge signature.

In contrast to the marine signature, the signature of the last glaciations has not been diluted at a distance of more than two meters from fracture zones allowing recharge fluid circulation. Although glacial water may exchange solutes with the microporosity at a local scale, it did not have time to equilibrate through diffusion with the surrounding rocks. Major fractures allow water circulation and microporosity acts as a reservoir for solutes. If water circulation is slow, the water in the fracture may equilibrate through diffusive processes with solutes from the microporosity immediately around the fracture without modifying the water signature^16^,^55^. However, the glacial signature will remain much more heterogeneously distributed than the much older marine signature.

The correlation between recharge temperature and chloride concentrations also supports this interpretation even though these parameters are related to two distinct processes (marine transgression and glacial melting). It shows that the last major circulation events were triggered by conditions extremely different to present conditions. Density-gravity-driven circulation is required to inject marine solutes at depth and the glacial recharge may be related to a much lower sea level and much deeper piezometric levels in the continent. Such lowering of the water table could have allowed efficient recharge as it limits the river network extension and increases the distance with the watershed surface. As the groundwater could not be discharged into the river network deeper flow i.e. regional loops could have been promoted. The most saline and deepest samples present the highest salinity and the coldest recharge temperature, as the original zones of marine signature, and also reflect the most rapid and active circulation of post-glacial water with high excess air and low recharge temperatures.

Recent modeling of groundwater infiltration during the glacial cycle in Canada indicated that major infiltration occurred below the glacier during the ice sheet progression whilst it remained limited in the permafrost areas^56^. A transient groundwater discharge regime also seemed to dominate during the deglaciation period. Geochemical observations may however indicate more complex processes in the periglacial areas^27^. The Armorican basement is considered to have been a discontinuous periglacial permafrosted area during the last glacial cycle^49^. The evidence of ubiquitous glacial water about 17,000 yrs ago rather indicates a continuous permafrost and a massive breakthrough at the onset of the glaciers retreat before the generalized deglaciation.

### Modern recharge

Although the salinities are not high, in comparison to the original seawater, the homogeneous preservation of salinity throughout the Armorican basement and the clear increase with depth indicates the limited downward circulation of modern groundwater. Circulation loops are mainly constrained by potential discharge zones. The depth of groundwater penetration is limited both by the surface hydrological network and the high sea level (as compared to during the glacial period). The non-existence of high elevation recharge zones within the Armorican basement, which could have flushed the deep saline fluids, has allowed the signatures of both the marine transgression and the glacial circulation events to be preserved.

## Conclusion

Investigation of a large database of chloride concentrations in the groundwater of hard rock aquifers in the Armorican basement in western France, revealed higher chloride concentrations below 100 m asl than could be expected from meteoric and anthropogenic sources. Twelve sites presenting chloride concentrations higher than 80 mg/L were investigated for isotopic, geochemical and dissolved gases analyses.

These data are interpreted as reflecting: (1) Introduction of marine seawater during the last transgressions 5.3 to 2 M yrs ago through gravity- and density-driven infiltration. This introduction was followed by diffusion of the marine solutes throughout the entire hard rock domain at the scale of several hundred meters, and exchanges with the rock microporosity. (2) Rapid recharge of meteoric water immediately following the last glacial maximum. Glacial water circulated in the main active fluid circulation zones. This water may have equilibrated with the surrounding rocks during the last 17,000 yrs thus allowing a single sample to contain both marine solutes and meteoric glacial solutes. (3) Following these two phases, modern groundwater circulation seems to be depth-limited as no dilution of these signatures has occurred.

This study indicates that marine transgression leads to a generalized introduction of seawater into hard rock aquifers. Diffusion allows the marine signature to be present throughout the area. It should thus be quite a common component of groundwater in hard rock aquifers. Conversely glacial recharge seems a rapid event, related to a restricted period related to the breakthrough of the permafrost. It indicates that glacial water may circulate even in permafrosted areas, at least during short time periods. The presence of a glacial signature throughout the Armorican basement indicates that glacial water, probably permafrost melt water, has a huge impact on groundwater even if it represents a time-limited event.

As compared to saline fluids in basement elsewhere, the Armorican fluids agree with the overall depth-salinity relationship^41^. The most striking difference is the relatively shallow depth of these fluids, probably induced by the lack of relief, and the relatively recent age of the transgressions. These two points have allowed both the marine and glacial signatures to be preserved, however the scenario described should have a large applicability, at least as test hypothesis, in crystalline rocks worldwide. The Armorican saline fluids thus confirm that fluid signatures in basements may record the various signatures of geologic and climatic events. They can be considered as archives of “crisis” events at the geological scale, which are recorded in the basement aquifers. It also emphasizes the role of fault-zones in the fluid transfer at depth. Our results also indicate that the extent of hydrogeological circulation of fresh water has been limited to less than 50 to 100 m during the last 17,000 yrs. Thus our findings emphasize the high sensitivity of groundwater resources to global climate changes as well as to anthropogenic pressure. They provide time-constraints for groundwater modeling under various climatic scenarios.

## Additional Information

**How to cite this article**: Aquilina, L. *et al.* Impact of climate changes during the last 5 million years on groundwater in basement aquifers. *Sci. Rep.* **5**, 14132; doi: 10.1038/srep14132 (2015).

## Supplementary Material

Supplementary Information
