# Supplementary material for: Impact of climate changes during the last 5 million years on groundwater in basement aquifers
Source: Sci Rep. 2015 Sep 22;5:14132. doi: 10.1038/srep14132 (PMC4585752; doi:10.1038/srep14132)

Impact of climate changes during the last 5 million years on groundwater in basement aquifers

Luc Aquilina[1], Virginie Vergnaud[1], Antoine Armandine Les Landes[1], Hélène Pauwels[2], Philippe Davy[1], Emmanuelle Pételet-Giraud[2], Thierry Labasque[1], Clément Roques[1], Eliot Chatton[1], Olivier Bour[1], Sarah Ben Maamar[3], Alexis Dufresne[3], Mahmoud Khaska[4], Corinne Le Gal La Salle[4], Florent Barbecot[5].

Supporting information

Figure S1: Evolution of the sea level in the Armorican Massif (Data from Ardenbol et al., 1998)

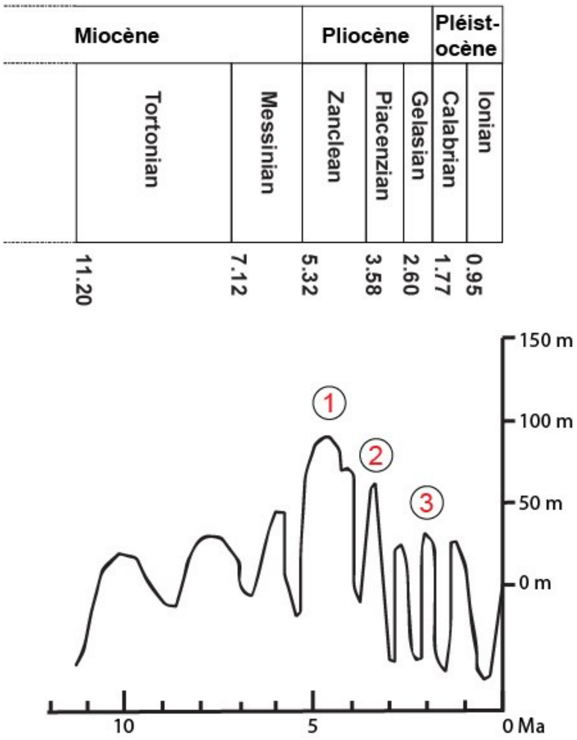

Table S1: Characteristics of the sampling points

Sample	x L2e (m)	y L2e (m)	alt. (m ASL)	main geology	dist. Sea (km)	Well depth (m)	Sampling depth (m)	Screened section
**Non saline fluids in the upper aquifers**								
Ploemeur F9 (100m)	167650	2321130	35	Micaschist and granite (contact zone)	5,5	100	50	44-96
Quessoy Captage (10m)	231467	2392595	60	Granitic sands	9,0	10	wss	~3-10
Betton Pz3 (13m)	303307	2362533	50	Brioverian schist	44,0	13	10	5-13
Kerbernez (10m)	117784	2346656	30	Granite		10 to 20	wss	10-20
St Brice (<10m)	325110	2386900	120	Metamorphic schists		< 10	wss	3-10
**Mixed fluids**								
Ploemeur F35 (133m)	168107	232101	25	Micaschist and granite (contact zone)	5,5	133	25	21-132
Ploemeur F11 (100m)	168150	2321160	26	Micaschist and granite (contact zone)	5,5	100	85	56-96
Saint-Fulgent (121m)	332185	2212410	93	Brioverian schist	58,0	121	wss	34-78
Cossé-le-vivien (55m)	354130	2331710	65	Brioverian schist	119,0	55	wss	5-48
Ploemeur F38 (150m)	168027	2321218	30	Micaschist and granite (contact zone)	5,5	150	85	19-150
Quessoy Forage (60m)	231492	2392615	60	Cadomian granitoids	9,0	60	40	22-66
La Garnache (72m)	282850	2218750	30	Metamorphic rocks	18,5	72	48	13-67
Bubry F3 (100m)	185984	2338056	50	Mylonitic rocks	33,0	100	35	48-100
Teillais F1 (137m)	314886	2319402	40	Schists and sandstone	82,0	137	80	20/30 - 137
Teillais F2 (137m)	314860	2319360	45	Schists and sandstone	82,0	137	80	20/30 - 137
Lanleff FE19 (118m)	204347	2423260	30	Metamorphic rocks	8,0	118	wss	20/30 - 118
Lanleff FE20 (100m)	204299	2423282	26	Metamorphic rocks	8,0	100	wss	20/30 - 118
**Deep saline fluids**								
Bubry F6 (100m)	186175	2337902	44	Michaschist and mylonitic rocks	33,0	100	36	48-100
Bubry F5 (100m)	186130	2337816	44	Micaschist	33,0	100	60	42-100
Betton Pz6 (85m)	303301	2362537	50	Brioverian schist	44,0	85	80	34-81
Cinergy (675m)	297712	2346124	39	Tertiary bassin and Brioverian schist below 400m	65,0	675	85	open hole 400-675

wss: Whole screened section (drinking water production)

Sample origin and water-rock interaction

The rationale for sampling was to collect deep saline samples and, as far as possible, shallow sample at the same site in order to compare saline samples to modern and shallow non-saline samples. It was however not always possible to have acces to shallow groundwater in all the sites. Two shallow modern groundwater samples from research monitoring sites (Kerbernez and St Brice en Coglès sites in Figure 1 and Table S2) have been added to the groundwater data set. These samples are characteristics of Brittany shallow groundwater. A PCA was carried out on the chemical analysis (Figure S2). The major variance (PC1: 47%, PC2 : 17%) is explained by an axis which opposes O_2, NO_3, Recharge temperature, CFCs (not shown) and Eh on one side and pH, Cl and Ar on the other side. This axis thus clearly distinguishes the most saline, old, reduced and basic with low recharge temperature samples from the modern, non-saline, oxidized and acidic samples with high nitrate concentrations. The first group includes the most saline Cinergy, Betton (Pz6) and Bubry (F5 and F6) samples whilst the second one includes the Ploemeur (F9) and Betton (Pz3) samples as well as the two shallow modern groundwater samples (Kerbernez and St Brice). Although sampled close to the surface, the Quessoy (captage) sample presents denitrification processes that lead to characteristics closer to deeper samples. All other samples represent intermediate situations some of them being close from the most modern end-member (Bubry F3, Ploemeur F35), other being much closer from the most saline samples (La Garnache, Lanleff, Cossé Le Vivien).

Interestingly, some of the moderately saline mixed samples, show high Ca, Sr, SO_4 and DIC concentrations that separates them from the most saline samples in the PCA. These high concentrations can be related to water-rock interaction processes. Silicate dissolution which may occur during long-term water rock interaction may release cations and sulphur minerals such as pyrite dissolution may account for SO_4 high concentrations. The Na/Cl ratios higher than seawater also support extensive water-rock interaction processes in these groundwater. The distinction between these samples and the most saline ones is probably related to the fact that salinity and water-rock interaction processes are decoupled. Water-rock interaction processes occurred after salinization/dilution processes leading to Cl concentrations close to the ones measured today in the samples. Within more dilute samples, cation sources create thus a higher variation, as compared to Cl, than in the most saline samples. As the characterization of the water-rock interaction processes is not the scope of the paper, we thus focus on the elements that are less influenced by water-rock interaction such as Cl, Br and to a less extent B and SO_4 to investigate the origin of the saline fluids.

Figure S2: Statistical analysis of the samples geochemistry

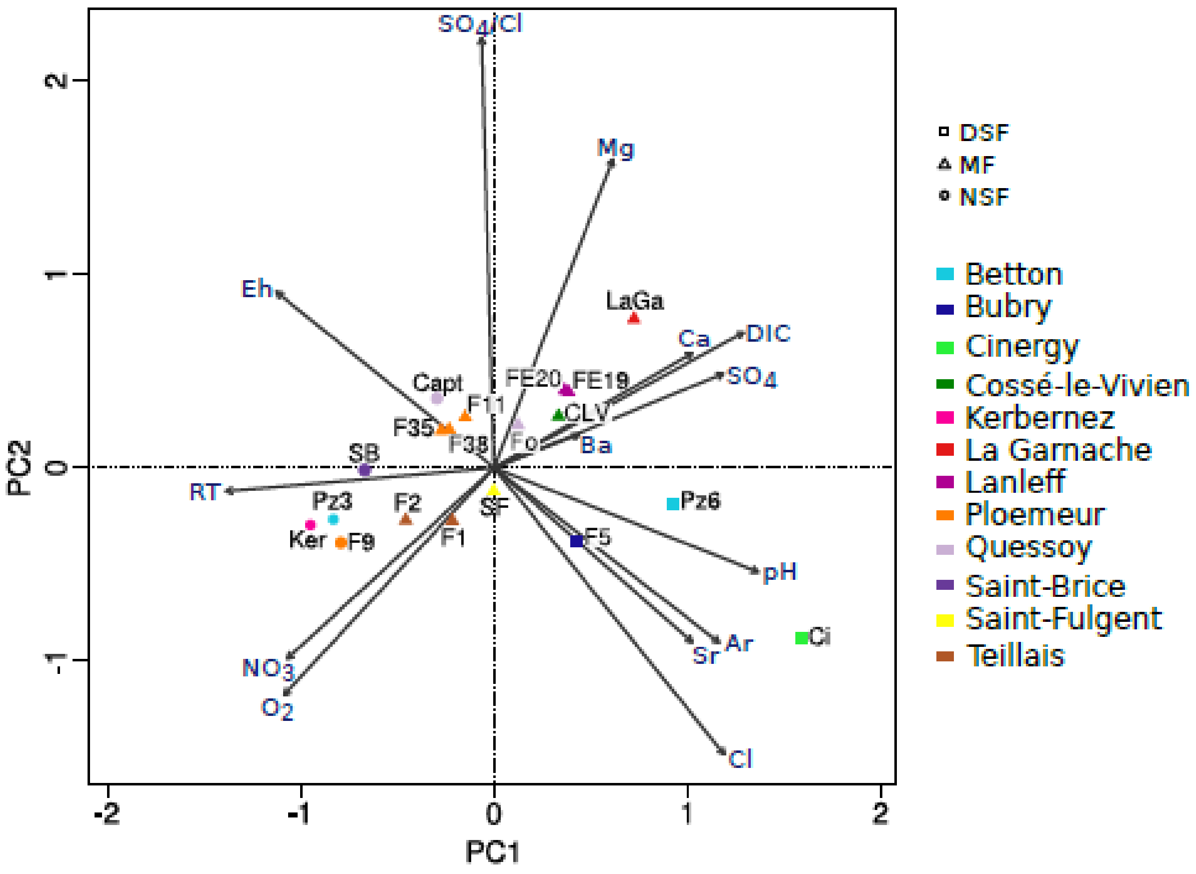

DSF: deep saline fluids / MF: mixed fluids / NSF: non saline fluids in the shallow aquifers

Mixings section

(1) Influence of modern groundwater. The saline fluids result from a complex mixing history that is somewhat difficult to decipher. As the saline samples are located far from the sea-coast and present reduced conditions they are thought to represent isolated parts of the aquifers. These samples contain almost no nitrate and CFC concentrations close to or below the detection limit (2). From the CFCs and nitrate content a range of mixing of the saline samples by modern surface fluids has been computed (the three CFC and nitrate concentration were used, the value in Table S1 correspond to the mean of the four values). On the basis of regional studies (1), we assumed deep saline fluids do not contain any anthropogenic tracers (mean regional NO_3 concentration in deep wells is < 1 mg/L) and considered the local CFC and NO_3 concentrations in shallow wells in the same site. Except for the Ploemeur site which is an intensively pumped site, most of the mixing rates are below 4.5 % (Table S2). For the three most saline sites (Bubry, Betton and Cinergy), mixing rates are below 4% for Bubry F3 and F6 and 1.6 and 1.4 % for Betton and Cinergy samples. Such mixings result from extremely slow renewal rates of these fluids or slight contamination during sampling.

(2) Seawater component. In table S2 are also presented the percentage of seawater necessary to explain the high salinity of the fluids. Percentages of seawater remain low, below 1% for most of the fluids. For the three more saline sites (Bubry, Betton, Cinergy) they are of 0.6 to 2.9 (Bubry), 3.85 (Betton) and 6.25 (Cinergy). Our hypothesis assumes a strong dilution of the paleo-seawater by glacial fluids. The very high content of solutes in seawater as compared to aquifer groundwater explains that the marine signature of Cl, Br, SO_4 and B has been preserved although saline fluids have been strongly diluted (see Fig. S4). Even a dilution of seawater by meteoric fresh water degree of 90% only induces a 0.5‰ shift of the mixing $\delta^{34}S$.

(3) Dilution of the seawater component by glacial fluids. As mentioned above, the saline fluids contain almost no modern aquifer groundwater. The low percentages of seawater in the saline samples have almost no effect on the oxygen and deuterium ratios, which explains that the waters have a meteoric signature for the stable isotopes. However, a part of aquifer groundwater older than 60yrs cannot be distinguished from glacial meteoric water. Only the oxygen-deuterium shift and the recharge temperature presented in the next section indicate the glacial signature. The percentage of glacial water versus non glacial fresh groundwater cannot be determined. As mentioned in the previous section, a strong dilution effect is consistent with the preservation of the marine signature of various solutes or isotopes, due to the high seawater concentration. For the ^{14}C, on the contrary, the low carbon content of seawater only slightly affects the ^{14}C content of the glacial and/or meteoric fluids (10% of seawater induces a lowering of 2% of the ^{14}C content).

The test of the various mixing hypothesis is consistent with the scenario described in the paper. A residual seawater component of a several percents allows the marine signature of ^{36}Cl, ^{11}B, Cl/Br, ^{34}S to be preserved. A strong dilution by glacial fluids will provide a meteoric signature for the stable O and D of water as well as ^{14}C ages consistent with the glacial dilution period.

Stable isotopes and noble gas

O and D measurements are provided in Table S3. Except the most saline Cinergy sample (Cl = 1,208mg/L), all the samples plot along the global meteoric water line, defining a potential

local meteoric water line (Figure S3A). This results demonstrate that the water has a meteoric origin which agrees with the low percentage of seawater contained in the samples (Tab. S2). However, when compared to the signature of the modern surface aquifer at the same place, the saline samples show a shift towards lower O and D values. To enlighten a potential temperature effect, this difference to the local surface reference has been computed. For the Cinergy sample, the shift is the difference between the fluid collected at the end of drilling (drilling fluid made of local tap water) and the final saline fluid produced after pumping.

The deuterium shift is plotted against the recharge temperature deduced from noble gases in Figure S3B. A good correlation is observed between these two parameters. For oxygen, the correlation is a bit less clear (R^2=0.57).

A recharge temperature has been deduced from the Ne and Ar content of the saline fluids. Although less precise than using a larger number of noble gases, the robustness of this computation has been tested. Furthermore, a more thorough analysis of all noble gases was carried out (mass spectrometry, East Anglia Laboratory) for one of the most saline fluid (Betton Pz6 sample, Cl = 747mg/L) and confirmed the noble gas recharge temperature obtained using Ne and Ar analysis.

---

1. Aquilina, L. et al. (2012) Nitrate dynamics in agricultural catchments deduced from groundwater dating and long-term nitrate monitoring in surface and ground waters. *Sci. Total Environ.***435**, 167-178.

2. Labasque T, Ayraud V, Aquilina L, Le Corre P (2006) Dosage des composés chlorofluorocarbonés et du tétrachlorure de carbone dans les eaux souterraines. Application à la datation des eaux. *Ed Géosciences* Coll Cahie:51 p.

3. Mook (1980) 14 C in *Handbook of Environmental Isotope Geochemistry*, ed Fontes PF& JC (Elsevier, New York).

4. Fontes JC (1992) Chemical and isotopic constraints on C-14 dating in *Radiocarbon after four decades: an interdisciplinary perspective*, eds Taylor R, Long A, Kra R (Univ. Californai Lake Arrowhead), pp 242–261.

5. Gillon M et al. (2009) Open to closed system transition traced through the TDIC isotopic signature at the aquifer recharge stage, implications for groundwater 14C dating. *Geochim Cosmochim Acta* 73:6488–6501.

6. Fontes J-C, Garnier J-M (1979) Determination of the initial 14 C activity of the total dissolved carbon: A review of the existing models and a new approach. *Water Resour Res* 15:399–413.

7. Phillips FM (2000) in *Environmental Tracers in Subsurface hydrology.*, eds Cook P, Herczeg AL (Kluywer Academic Publisher, Boston), p pp 299 – 348.

8. Argento DC, Stone JO, Keith Fifield L, Tims SG (2010) Chlorine-36 in seawater. *Nucl Instruments Methods Phys Res Sect B Beam Interact with Mater Atoms* 268:1226–1228.

9. Vigneresse JL, Cuney M, Jolivet J, Bienfait G (1989) Selective heat-producing element enrichment in a crustal segment of the mid-European Variscan chain. *Tectonophysics* 159:47–60.

10. Dabard MP, Peucat JJ (1998) Les métasédiments de la série du Pouldu: Etude géochimique et isotopique.

11. Dabard MP, Peucat JJ (2001) Les métasédiments de Bretagne sud. Etude géochimique et isotopique.

12. Roques C, Aquilina L, Bour O, Maréchal JC, Dewandel B, Pauwels H, Labasque T, VergnaudV, Hochreutener R (2014) Groundwater sources and geochemical processes in a cristalline fault aquifer. *J. of Hydrology **519**, 3110-3128.*

13. Ladouche B, Cherry L, Petelet-Giraud E (2004) Contribution à la caractérisation des états géochimique de reference des eaux souterraines. BRGM Report RP53025.

Table S2 : Chemical analyses of the saline fluids from the Armorican basement

Sample	pH	Eh (mV)	O_2 (mg/L)	Cl (mg/L)	Br (mg/L)	B (µg/L)	SO_4 (mg/L)	SO_4/Cl	Cl/Br	NO_3 (mg/L)	DIC (mg/L)	Ca (mg/L)	Mg (mg/L)	Na (mg/L)	K (mg/L)	Sr (µg/L)	Ba (µg/L)	CFC-12 pmol	CFC-11 pmol	CFC-113 pmol	Ne 10^{-8}mol/L	Ar 10^{-5}mol/L	Recharge T°C	% Seawater	% Mixing with modern gw
**Non saline fluids in the upper aquifers**																									
Ploemeur F9 (100m)	6,00	179	6,0	47	0,2	30	12	0,25	210	45,3	3,9	7,7	7,1	35,9	3,8	74,3	39,9	5,40	6,50	0,38	0,98	1,80	9	0,0	
Quessoy Captage (10m)	6,10	102	0,1	40	0,2	18	68	1,72	248	1,3	11,3	20,4	7,5	25,2	2,3	99,8	24,2	15,95	2,40	0,08	1,20	1,95	10	0,0	
Betton Pz3 (13m)	5,90	91	6,5	46	0,1	12	22	0,47	369	49,6	11,4	23,0	14,0	23,0	1,0	83,5	16,5	2,20	3,80	0,29	0,97	1,51	12	0,0	
Kerbernez (10m)	5,60	180	5,4	32	0,1	22	18	0,56	291	62,0	3,2	11,4	8,9	22,6	2,5	105	25,0	2,44	4,81	0,45	1,04	1,70	11	0,0	
St Brice (<10m)	5,63	131	3,9	24	0,05	23	26	1,06	483	30,9	20,3	14,3	6,8	16,8	4,0	0,1	21,2	2,21	4,03	0,46	1,29	1,88	11,5	0,0	
**Mixed fluids**																									
Ploemeur F35 (133m)	5,90	140	2,4	73	0,3	22	71	0,96	230	1,9	21,1	18,8	17,7	54,0	4,2	132,7	11,8	12,00	8,40	0,30	1,53	2,05	9	0,19	24,6
Ploemeur F11 (100m)	6,30	143	1,8	74	0,3	48	88	1,18	251	1,0	15,2	19,5	18,3	60,2	4,7	141,9	15,4	1,50	0,30	0,06	1,41	2,12	7	0,19	9,3
Saint-Fulgent (121m)	6,90	-3	0,9	79	0,3	9	13	0,16	254	3,1	29,6	25,4	15,5	58,6	1,1	132,2	14,4	0,40	0,15	0,03	1,57	2,12	8	0,22	10,0
Cossé-le-vivien (55m)	6,90	-20	bdl	80	0,3	28	108	1,22	341	5,9	40,4	80,9	12,2	48,6	1,9	722,9	11,4	1,20	0,15	0,04	1,04	1,90	8	0,22	19,0
Ploemeur F38 (150m)	6,50	166	2,0	87	0,4	70	92	1,05	226	7,4	15,6	20,6	16,5	56,6	3,8	121,8	11,2	0,65	0,20	0,04	1,27	2,01	8	0,26	9,6
Quessoy Forage (60m)	6,60	89	bdl	96	0,4	86	120	1,25	266	1,1	24,8	12,8	8,1	122,1	5,7	103,8	44,0	3,30	0,15	0,02	1,64	2,29	5	0,31	16,8
La Garnache (72m)	6,90	-1	0,2	96	na	159	144	1,50	-	0,5	58,5	68,5	42,4	93,1	3,5	421,2	71,2	0,50	0,10	0,04	1,25	2,10	5	0,31	4,4
Bubry F3 (100m)	8.3	-49	0.4	119	0.44	79	5.0	0.04	270	2.4	17.3	3,3	6,7	93,0	1,8	48,4	1,0	0.3	0.2	0.05	1.10	1.88	10	0.61	4.6
Teillais F1 (137m)	5,70	-20	1,4	113	0,4	47	43	0,38	276	13,0	3,8	17,8	6,0	71,4	3,3	239,9	40,3	1,30	1,35	0,09	1,76	2,25	8	0,40	6,3
Teillais F2 (137m)	5,45	127	4,2	142	0,5	53	46	0,32	279	9,2	5,2	21,4	6,8	88,5	3,5	307,4	40,0	1,70	2,00	0,11	1,72	2,15	10	0,55	3,6
Lanleff FE19 (118m)	7,50	146	bdl	142	0,5	64	138	0,97	283	5,7	33,7	60,6	23,4	83,4	2,6	188,2	45,8	2,00	0,50	0,30	1,26	2,13	5	0,55	3,2
Lanleff FE20 (100m)	7,55	160	bdl	144	0,5	na	139	0,96	281	5,6	33,1	60,8	23,4	83,4	2,7	185,5	45,0	1,70	0,45	0,33	1,20	2,07	5	0,56	3,4
**Deep saline fluids**																									
Bubry F6 (100m)	na	na	na	347	1,2	373	40	0,11	293	0,1	17,4	31,9	2,7	255,5	6,4	602,7	28,0	na	0,20	0,05	1,15	1,92	10	1,63	1,2
Bubry F5 (100m)	7,50	91	1,5	565	1,9	410	41	0,07	300	11,2	21,3	86,9	11,3	630,1	14,6	1669,8	84,6	na	na	na	1,04	1,87	8	2,78	12,0
Betton Pz6 (85m)	7,20	-35	2,7	746	2,6	561	307	0,41	286	bdl	30,7	59,0	30,0	649,6	12,0	1553,4	19,6	0,05	0,10	0,03	1,58	2,31	5	3,73	1,6
Cinergy (675m)	10,00	-208	0,1	1208	3,9	624	166	0,14	313	bdl	53,5	39,4	4,9	934,0	23,1	990,0	28,3	2,30	0,05	0,08	4,98	3,60	1	6,17	1,4

Table S3 : Isotopic analyses of the saline fluids from the Armorican basement

Sample	$\delta^{34}S$ (‰) (SO_4)	$\delta^{18}O$ (‰) (SO_4)	$\delta^{2}H$ (‰) (H_2O)	$\delta^{18}O$ (‰) (H_2O)	$\delta^{11}B$ (‰) (H_2O)
**Non saline fluids in the upper aquifers**					
Ploemeur F9 (100m)	16.4	3.9			44.5
Betton Pz3 (13m)	11.0	4.4	-39.5	-6.34	26.5
St Brice	8.0	7.8	-39.7	-6.1	23.5
**Mixed fluids**					
Ploemeur F35 (133m)	10.8	7.5	-31.4	-5.40	34.3
Ploemeur F11 (100m)	5.9	8.0	-31.2	-5.31	35.5
Saint-Fulgent (121m)	3.5	7.8	-36.6	-5.70	35.3
Cossé-le-vivien (55m)	3.6	1.2	-41.6	-6.30	33.9
Ploemeur F38 (150m)	14.8	11.2	-32.4	-5.41	36.6
La Garnache (72m)	16.7	11.1	-34.3	-5.30	16.2
Lanleff FE19 (118m)	11.1	4.4			24.9
**Deep saline fluids**					
Bubry F5 (100m)	24.2	13.6	-37.4	-5.90	8.7
Betton Pz6 (85m)	22.3	16.0	-42.7	-6.82	33.9
Cinergy (675m)	25.0	15.2	-35.3 / -34.0	-4.70 / -4.60	40.7

Table S4 : 36-chlorine measurements

	Total Cl (mmol/L)	36Cl (10^{-15})	Cl36/Cl35 (10^{-14})	Uncertainty Cl36/Cl35 (%)
Ploemeur F38	2.507	1.27	5.07	5.02
Betton PZ6	20.986	5.27	2.51	7.17
Cinergy	26.479	6.45	2.44	7.19

Table S5 : 14-carbon measurements and residence time computations

Sample	$\delta^{13}C$ (‰ vs PDB)	$A^{14}C$ (pmc)		pH	HCO_3 (mmol/L)	$\delta^{13}C$ at equilibrium (‰ vs PDB)	Age - Mook model (yr BP)	Mixing with modern surface fluids (%)	Age corrected for mixing (yr BP)
**Non or slightly saline fluids in the upper aquifers**									
Ploemeur F9	-20.83	95.0	± 0.2	5,6	0,68	-21.0	Modern		
Ploemeur F11 (85m)	-18.46	77.8	± 0.2	5,4	1,27	-18.2	Modern		
Ploemeur F37 (85m)	-19.06	82.1	± 0.2	6,3	1,58	-22.3	1600		
Ploemeur MF2	-19.08	84.7	± 0.2	6	1,23	-20.7	Modern		
Saint Brice F3	-18.27	81.71	± 0.3	5,8	1,44	-19.1	Modern		
**Most saline fluids**									
Ploemeur F38 (80m)	-17.64	61.1	± 0.2	6,6	1,78	-22.7	4300	15	6500
Betton P26 (85m)	-15.93	15.6	± 0.1	7,2	2,56	-23.6	16600	1	17100
Cynergi (400m)	-18.31	26.5	± 0.15	10	4,46	-27.1	16000	5	17600

^{14}C has been measured in three saline samples and in non or slightly saline samples at depth ranging from 50 to 85m in two sites. The carbon isotopic composition (^{13}C, ^{14}C) of total dissolved inorganic carbon (TDIC) in groundwater is acquired mainly during transit in the unsaturated zone by exchanges with soil CO_2 and the carbonate matrix (5). For all samples, the calculated $\delta^{13}C$ of the gaz in equilibrium with the water TDIC do not evidence any equilibration with the carbonate matrix in the saturated zone which provides a good confidence to the age computation. The high pH of the Cinergy sample induces a very low equilibrated $\delta^{13}C$ but has limited consequences on the age computation. Considering Fontes (1992), the appropriate adjustment model corresponds to the one proposed by Mook (1980) (3, 4). It has been applied to all data (Table S5). A correction has been introduced using the mixing with surface modern groundwater defined from NO_3 and CFC concentrations. The activity of the Ploemeur F9 sample (highest ^{14}C activity) has been used as a modern end-member to correct the A_0 activity. No correction has been introduced for the $\delta^{13}C$ or the pH as the mixing percentages are low. Ages have also been computed using an equilibrating model (6) showing a relatively low uncertainty, below 1,000yrs.

Figure S4 : 36-chlorine versus 1/Cl

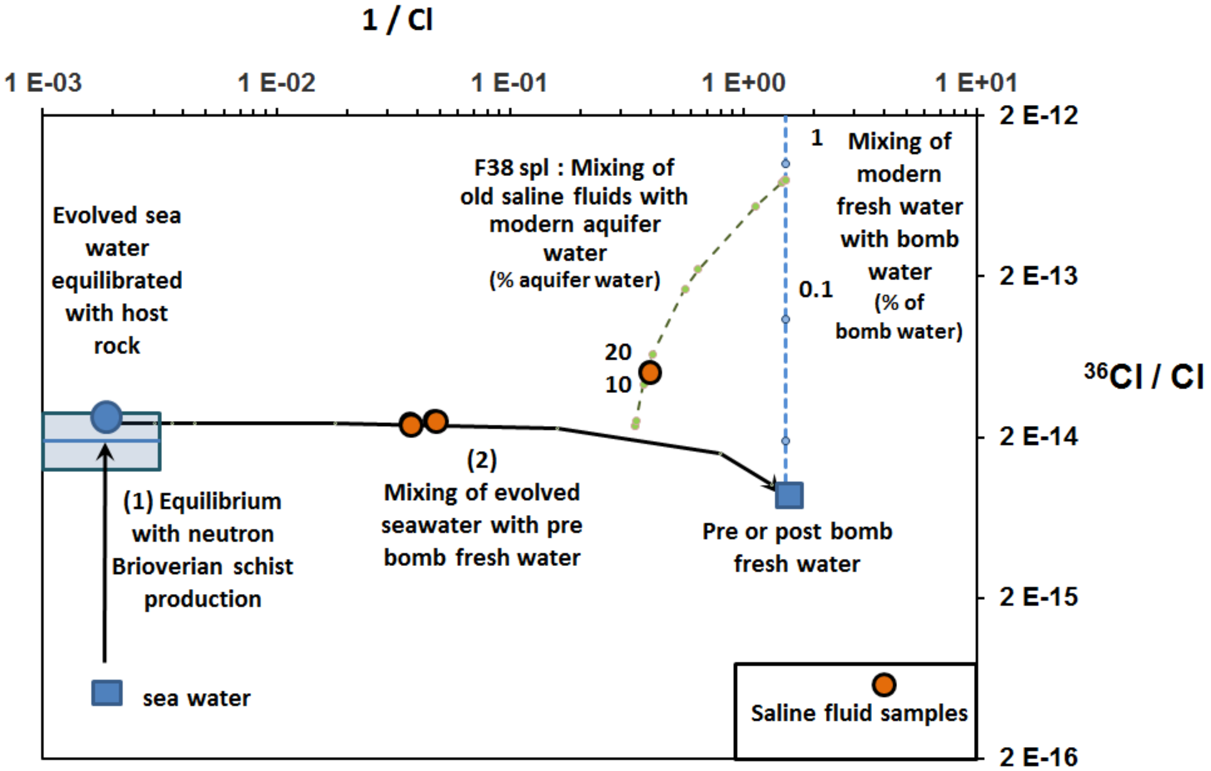

Modern (i.e. post bomb) fresh waters have $^{36}Cl/Cl$ in the range 2 to 6 10^{-15} (7). Surface aquifers (weathered part of the aquifer, 0-30m) have a mean residence time of 15-20yrs and annual renewal rates ranging from 2 to 15% [33, 34]. Thus it might be expected that these aquifers only contain low amounts of ^{36}Cl. The dotted line in Fig. S4 represents the potential mixing line of modern aquifer waters including various amounts of bomb ^{36}Cl and low Cl content.

Saline samples have $^{36}Cl/Cl$ above those of post-bomb fresh waters and present CFC concentrations below or close to the detection limit. They cannot represent modern aquifer waters including a slight bomb component as they are older than 50yrs and much more saline.

We interpret these samples as resulting from seawater introduction in the basement long-time ago. Seawater has initially a low $^{36}Cl/Cl$ ratio (8) and then equilibrates with the neutron production flux of the rock (arrow 1 in Fig. S4). The secular equilibrium value (Rse) has been defined from the U and Th concentration in the Brioverian schists of the Armorican basement (9–11) which contains the saline fluids and represents a large part of the Armorican basement (mean U and Th : 3.4 ± 1.4 and 10.4 ± 2.5 ppm) which provides a Rse range of 1.09 10^{-14} to 2.16 10^{-14} and a mean of 1.62 10^{-14}. The Ploemeur schist which is close to a granite has slightly higher U and Th concentrations (9 and 16 ppm) and a Rse of 2.69 10^{-14}. These values are represented in Fig. S4 as the box on the left hand side. The saline sample presents values that are close to the highest range, which suggest that they have reached the secular equilibrium and thus present residence time higher than 1 10^{6}yrs.

Then, these fluids are strongly diluted by glacial fluids which are considered as an equivalent of pre-bomb meteoric fresh water. As these fluids only contain very low Cl concentrations, they almost have no effect on the $^{36}Cl/Cl$ ratio unless mixing reaches more than 99% of fresh water.

Finally, the higher $^{36}Cl/Cl$ ratio of the Ploemeur F38 sample might result from the component of modern surface water which is present in this sample. Mixing of evolved seawater diluted

by glacial fluids with modern aquifer groundwater is sketched in Fig. S4. $^{36}Cl/Cl$ ratio of F38 sample might be explained by introduction of 10 to 20% of modern aquifer groundwater, which agrees with the 15% deduced from CFC and nitrate concentrations (Tab. S2).

Figure S3A: Stable isotopes diagram

Arrows relate surface samples to deep saline or mixed samples. St Brice en Coglès is a non-saline deep sample outside the transgression areas which however presents low recharge temperature (see further details in Roques et al., 2014) (12). Bubry surface sample from a close site (Ladouche et al., 2004) (13).

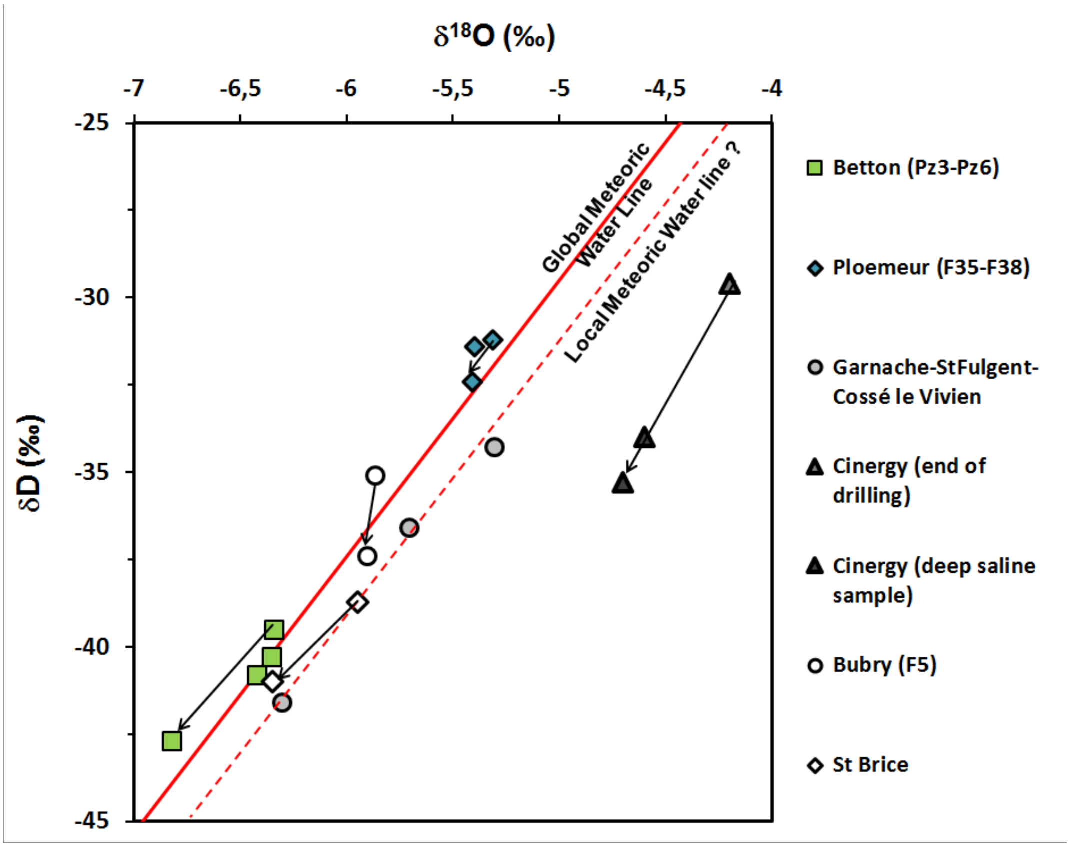

Figure S3B : Deuterium shift versus recharge temperature

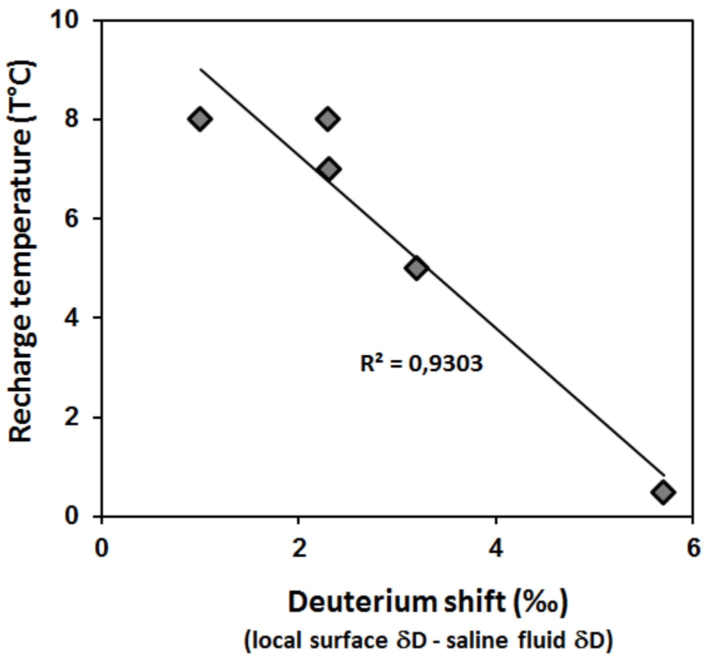

Supplement: Supplementary Information [file srep14132-s1.pdf]
